# Candlenut oil-induced sclerosing lipogranuloma of the penis: A case report

**DOI:** 10.1016/j.ijscr.2023.108673

**Published:** 2023-08-18

**Authors:** Adhitya Wisnu Mahadewa, Yustin Marinta, Prapanca Nugraha, Kiki Lukman

**Affiliations:** Department of Surgery, Faculty of Medicine, Universitas Padjadjaran, Perpetua J. Safanpo General Hospital, South Papua, Indonesia

**Keywords:** Case report, Foreign-body granuloma, Oil injection, Penis, Sclerosis

## Abstract

**Introduction and importance:**

Penile sclerosis lipogranuloma, a disease that occurs as a result of the body's reaction to lipid-based foreign substances, is a rare case with manifestations that can occur years after injection. Reactions that emerge can be disturbing to the point of causing functional impairment, so proper therapy needs to be done to restore and maintain penis function and prevent complications. Here, we present a case of penile sclerosis lipogranuloma that was treated surgically with a scrotal flap and V—Y plasty, including circumcision.

**Case presentation:**

We report here the case of a 19-year-old Asian male who came in with multiple, irregular, nodular masses in his penis after a candlenut oil injection that had been performed a year before presentation. An extensive excision and extraction of the penile lipogranuloma, including all areas invaded by oil injection, were performed. Then a scrotal flap and V—Y plasty were used to reconstruct the exposed penile shaft. The operative procedure was successful, and the patient experienced positive functional and aesthetic outcomes.

**Clinical discussion:**

Determining therapy for penile sclerosis granuloma becomes important to improve or restore normal penile function and for performance function. Therapy includes the complete removal of the substance and the affected part. The recommended reconstruction for the penile shaft is a scrotal flap with penile scrotal invagination and V—Y plasty.

**Conclusion:**

Proper treatment of the penis and its surroundings in cases of penile lipogranuloma is important to prevent further complications and maintain penile function.

## Highlights

• Penile sclerosing lipogranuloma is rare.
• Extensive excision and extraction of the penile lipogranuloma are important.
• Reconstruction is essential to restore and maintain penis function.
• Scrotal flap and V—Y plasty are choices for penile shaft reconstruction.

## 1 Introduction

Penile sclerosis lipogranuloma is a reaction of the body to foreign lipids, hydrocarbons (paraffin tumors), silicones, methacrylates, hyaluronic acid, or collagen patches. Increasing the size of the penis and improving the shape of the penis are usually the main goals of using injection materials such as liquid paraffin, vaseline, and others [1]. Mostly happening to men less than 40 years old, reactions usually occur one to two years after the foreign material is injected and may also be accompanied by chronic inflammatory sclerosis, which causes a decrease in function [2]. Manifestations can also be felt years after injection, such as foreign granulomas known as paraffinomas, which are associated with the amount of tissue damage [3]. The reaction is due to the fact that the material contained in the injected substance cannot be broken down or metabolized by the body because the body does not have the enzymes to break down lipids that come from outside the body [4,5]. Apart from the sequelae mentioned earlier, there is also an increased risk of penile cancer [6,7]. Factors such as the number of injections, composition, injection site, depth, and time since injection affect the clinical symptoms that appear. Usually, the symptoms that appear quickly after the injection are swelling, erythema, and edema of the skin of the penis. Symptoms that appear can be felt for years without any pain. Fibrosis will gradually occur, and then the penis will become hard, painful, and ulcerative. Later, the skin becomes sensitive at the injection site, often with a dark yellowish discoloration. Complications can occur weeks or years after the injection. A biochemical analysis is required to determine the composition and origin of the injected substance if the patient does not want to tell the truth [8]. Substances that have been injected can spread and move to other areas, including regional lymph nodes, due to the large amount and low viscosity of the injected substance [9,10]. The prepuce can store injectable substances and can cause phimosis to occur in uncircumcised patients. A biopsy is needed to differentiate granulomas from malignancies and determine surgical procedures. Diagnosis and treatment planning can be assisted by ultrasonography (USG) and magnetic resonance imaging (MRI). Soft tissue or surrounding body parts that are involved are best seen on an MRI. One of the medications is the use of steroids, but there are reports that state that patients who do not respond to steroids so that recurrences occur can only be treated with surgery [11]. Reconstructive surgery is the best treatment option when the removal of all granulomas by full excision is performed to prevent recurrent scars or skin necrosis [8,12,13]. Here we present a case of penile sclerosing lipogranuloma that was treated with one-stage surgical excision with a scrotal flap and V—Y plasty, including circumcision. The reporting of this case follows the SCARE criteria [14].

## 2 Case report

**Fig. 1:**
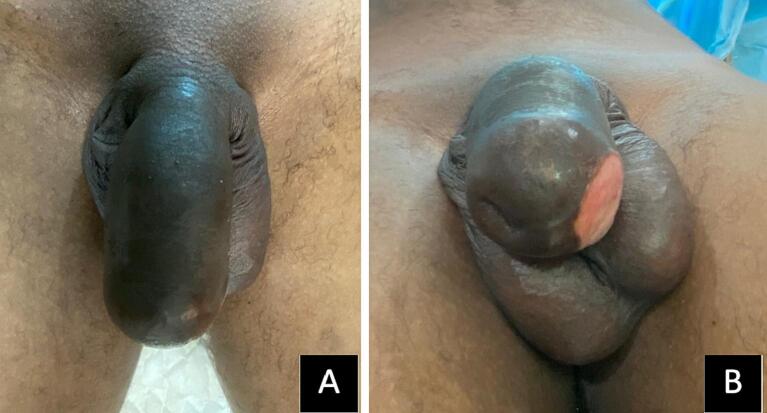
Penile sclerosing lipogranuloma; A. Blackish discoloration of the penis skin with multiple, irregular, nodular masses; B. Painless granulated ulcer on the glans of the penis.

A 19-year-old Asian male came with multiple, irregular, nodular masses in his penis after a hair oil or candlenut oil injection (Fig. 1). He underwent an oil injection for the purpose of enlarging his penis. Penile injections had been performed a year before presentation by some untrained, non-medical person. Two months prior, he felt the skin around his penis become darker, there was slight discomfort on his penis, and there was mild pain when an erection occurred. He did not have any symptoms of early ejaculation or erectile dysfunction. The pain diminished when resting. There was a history of oral medication and corticosteroid use one month before the consultation at the surgery polyclinic, but the discomfort persists. He did not have any surgical history or systemic diseases previously. Physical examination revealed that there was a small ulceration, slight fibrosis, and small, irregular, and multiple nodular masses without inguinal lymph node involvement. The preoperative laboratory findings, including urinalysis, complete blood count, and blood chemistry, were within the normal range. The imaging, including thoracic X-rays and ultrasonography of the abdomen, was normal. The MRI examination was not available at this primary general hospital. He was diagnosed with candlenut oil-induced sclerosing lipogranuloma of the penis (Soebhali classification 2) with a late reaction, including a painful and ulcerative penis. The indication for surgery in this case was pain and ulceration in the penile area. He was then scheduled for surgery. One general surgeon and one medical doctor at a primary general hospital carried out the surgery.

**Fig. 2:**
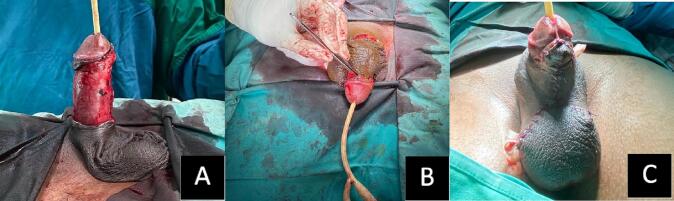
Intra-operative of one stage surgical excision with scrotal flap and V—Y Plasty; A. Complete excision of the sclerosing skin exposing the penile shaft; B. Then the exposed penile shaft was bent past the detached skin (penile scrotal invagination); C. The closure of the ventral part with an inverted Y-shape suture.

**Fig. 3:**
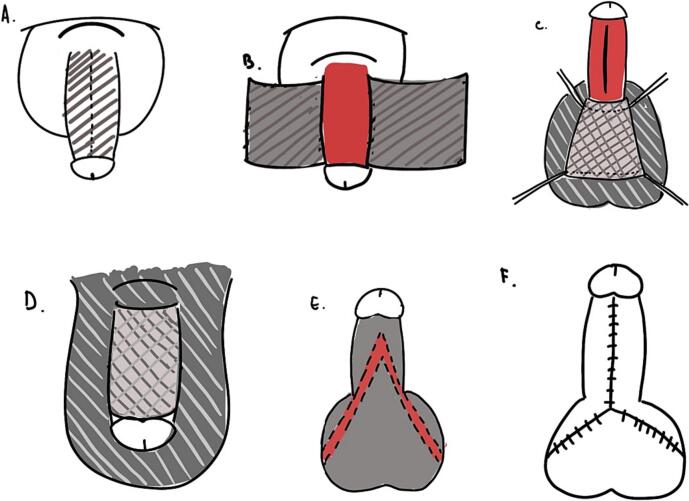
Schematic illustration of the scrotal flap and V—Y plasty; A. The sclerosing skin involved all skin covering the penile shaft; B. Complete removal of the sclerosed skin, exposing the naked penile shaft; C. Measurement of the exposed area to the scrotal flap using a four-way corner suture for precise measurement. The undermining scrotal flap was measured based on the length of the penis for adequate coverage; D. Penile scrotal invagination with the scrotal flap covering the dorsal penile shaft; E. Bilateral incision and undermining of the skin on the ventral part of the penile shaft (V incision); F. Closure of the skin with an inverted Y scrotal plasty.

Considering there was no involvement of the scrotal skin, the scrotal flap became the choice over a skin graft for a better cosmetic and functional outcome. The surgery performed was a one-stage lipogranuloma surgical excision with a scrotal flap and V—Y plasty, including circumcision. A careful wide excision and removal of the penile lipogranuloma using Metzenbaum and monopolar cautery was carried out in all areas affected by the oil injection, starting from the dorsal portion of the penile to the ventral portion, from the skin to the level of the Buck fascia, without damaging the dorsal neurovascular bundle. After the penile lipogranuloma was completely removed, an incision and undermining of the lower penoscrotal junction skin were performed with precise measurements of the penile length and width. Then the exposed penile shaft was bent past the detached skin (penile scrotal invagination). The exposed penile shaft was sealed, utilizing bilateral covering from the detached skin, and the detached skin was approximately sutured to the neck of the glans penis. The closure of the ventral part of the scrotal flap was completed with a bilateral horizontal incision (a V-shaped incision) and suturing up the subcutaneous tissue in an inverted Y shape with the placement of a modified surgical drain from a sterile glove (Fig. 2). The schematic illustration is shown in Fig. 3.

Postoperative management includes fluid maintenance, intravenous analgesics, and antibiotics. Removal of the drain and changing of the bandage were performed on postoperative day two, and the patient was discharged. He came to the polyclinic two times after surgery, at a two-week interval and a one-month interval, and showed no signs of complications such as surgical site infection or recurrence of pain or stiffness [[13], [14], [15], [16], [17]]. The operative procedure was successful, and the patient experienced positive functional and aesthetic outcomes; he denied any difficulty in erection or pain. His perspective on the procedure was, “I'm delighted that the pain disappears when an erection occurs.” His feedback was positive and showed satisfaction.

## 3 Discussion

The incidence of penile sclerosis lipogranuloma is very low, and there are few reports about it, with reactions that tend to occur one to two years after injection. Manifestations can occur early in the disease's progression or as late as years after injection [2]. The early reactions include swelling, erythema, and edema of the penis, which are often painless [8]. The late reactions can progress into a stiff, painful, ulcerative, and fibrotic penis. Other reactions that have been known include skin gangrene, local migration of the injected substance, blindness, embolization, sepsis, and even death [18,19].

Histopathological examination ensures an augmenting substance was injected, and it also tells the surgeon what kind of substances are being injected. The early result typically shows acute inflammation reactions circling and inside the vacuoles with reticular dermis densening [9,19,20]. Soft tissue or surrounding body parts that are involved are best seen on an MRI. The USG and MRI could aid in diagnosing and guiding treatment [21]. Conservative treatment is oral corticosteroids and the removal of the substance from the affected part with surgery. However, spontaneous regression after corticosteroids is rare, and in most cases, the regression results occur due to prior excision, either partially or completely, although it is proven that oral corticosteroids help reduce the inflammation [21,22]. The preferred treatment for penile sclerosis lipogranuloma is surgery [8,12,13].

The indications for surgery are pain at the granulomatous area or pain exacerbated by erection or intercourse, acquired phimosis due to edema of the uncircumcised area, lesion or ulceration of the injected area, functional disorder due to hardened skin, and poor cosmetic appearance due to a deformed penis [[23], [24], [25]]. Surgery is required to remove all granulomas to prevent recurrence, infected ulcers, or skin necrosis. For good surgical results, it is necessary to remove all granulomas. Reconstruction of the damaged skin can be done either with a graft or a skin flap. Harvesting a split-thickness or full-thickness skin graft from the abdominal or inguinal area is sufficient. However, using skin grafts for the reconstruction of the exposed area after removal of the granulomas has shown several disadvantages, such as contracture, a wider area of the surgical site (donor and grafted area), and a poor cosmetic result due to the difference in color. The skin flap shows more advantages compared to the skin graft. Skin flaps show less contracture, can keep the form of the penis, and show good cosmetic results [[25], [26], [27]].

There are two common flaps for reconstruction of the penile shaft: the Dartos Fascio-Myo-Cutaneous Flap (dartos flap) and the scrotal flap. The design and incision of the scrotal flap are simpler than those of the dartos flap. In this case, the author used the scrotal flap because of the abundant healthy scrotal skin after granuloma excision [[25], [26], [27]]. To reduce the risk of penis shortening, a scrotal incision is performed with the same length or longer as the penis. Shortening of the penis can also be treated with a simple V—Y flap. Drainage is done to prevent hematomas in the suprapubic area and scrotum [2].

As proposed by Soebhali [6,7], sclerosing lipogranuloma of the penis can be divided into:

1. Classification 1: Minimum wound, under one-third of the penis, without scrotal or suprapubic involvement.
2. Classification 2: Wound affecting the penile shaft greater than one-third of the penis, without scrotal or suprapubic involvement.
3. Classification 3: Wound affecting the penile shaft involving the suprapubic and scrotal areas of less than half
4. Classification 4: Wound affecting all the penis region, involving suprapubic and scrotal areas greater than half.

In this case report, the patient suffers from penile lipogranuloma, Soebhali classification 2. The scrotal flap was the best choice for reconstruction for this classification. Determining therapy for penile sclerosis granuloma becomes important to improve or restore normal penile function and for performance function. The right choice of therapy must also be done appropriately to prevent complications and worsening of the final outcome.

## 4 Conclusion

Penile lipogranuloma is a rare and serious complication from the injection of foreign substances into the penile. Surgical management is important for the removal of the granuloma, restoration of penile function, and cosmetics. The scrotal flap is a simple reconstruction surgery for the penile shaft with good cosmetic and functional results.

## Sources of funding

The author(s) received no funding for this case report.

## Ethical approval

The Hospital Ethical Committee approved this report with registered number 800/0801/RSUD-PJS/VII/2023 on July 20, 2023.

## Consent

The patient's written informed consent had to be obtained before this case report and the associated photographs could be published. The chief editor of the journal may evaluate a copy of the written consent upon request.

## Research registration

Not applicable.

## Guarantor

The guarantor of this report is the first author.

## Provenance and peer review

Not commissioned, externally peer-reviewed.

## CRediT authorship contribution statement

**Adhitya Wisnu Mahadewa:** Investigation, Data curation, Writing – original draft, Resources. **Yustin Marinta:** Investigation, Data curation, Writing – original draft, Resources. **Prapanca Nugraha:** Writing – review & editing, Project administration. **Kiki Lukman:** Conceptualization, Visualization, Supervision, Funding acquisition.

## Declaration of competing interest

The authors state that they are aware of no personal or financial conflicts that might have appeared to have an effect on the case reported.
